# Sublingual microcirculatory changes during transient intra-abdominal hypertension: a study in laparoscopic surgery patients

**DOI:** 10.1186/cc12149

**Published:** 2013-03-19

**Authors:** L Maddison, K Riigor, J Karjagin, J Starkopf

**Affiliations:** 1 University of Tartu, Tartu University Hospital, Tartu, Estonia

## Introduction

Microvascular alterations play an important role in development of organ failure [1]. It is not known whether increased intra-abdominal pressure (IAP) is associated with microcirculatory perfusion derangements. Our hypothesis was that transiently increased IAP is related to microcirculatory alterations in laparoscopic cholecystectomy patients.

## Methods

Sixteen patients (14 female, two male) who underwent laparoscopic cholecystectomy were studied. Sublingual orthogonal polarization spectral (OPS) imaging was used to detect microcirculatory function. OPS was done before surgery, at least 15 minutes after initiation of pneumoperitoneum and 1 hour after the end of pneumoperitoneum. The microcirculation cutoff value for vessels was 20 μm. Data are presented as medians with interquartile ranges.

## Results

Patient median age was 54 (39 to 63) years, ASA score 2 (2 to 3), BMI 29.7 (24.9 to 34.7), haemoglobin concentration 138 (133 to 142) g/l, and hematocrit 42 (39 to 43). IAP was held at 12.5 (12 to 13) mmHg, median duration of pneumoperitoneum was 45 (24 to 55) minutes. Median MAP was 86 (69 to 93), abdominal perfusion pressure (APP) 73 (57 to 81) mmHg during the pneumoperitoneum. Median fluid administration during anesthesia was 1,050 (1,000 to 1,400) ml. Altogether 448 microcirculation videos were taken. Interobserver variability was 24%. The following microcirculatory parameter values describe before, during and after pneumoperitoneum periods. Total vascular density was 19.4 (17.0 to 21.1); 18.5 (17.0 to 20.9); 19.3 (16.9 to 20.9) n/mm^2^. Perfused vessels density was 13.3 (10.9 to 15.2); 13.8 (8.9 to 18.0); 13.1 (11.0 to 16.0) n/mm^2^. Proportion of perfused vessels (PPV) was 61 (50 to 69); 64 (45 to 76); 60 (54 to 67)%. Microvascular flow index was 2.4 (2.0 to 2.5); 2.5 (2.0 to 3.0); 2.3 (2.0 to 2.9) and heterogeneity index was 0.8 (0 to 0.9); 0.6 (0 to 1.0); 0.6 (0 to 0.8). No significant differences in microcirculatory parameters were observed between time points. PPV was somewhat less than that of described in healthy volunteers (61% vs. 90%) [2].

## Conclusion

Microcirculatory alterations are mild during transient increase of intra-abdominal pressure in laparoscopic surgery patients.
